# Diagnosis and management of hypoglossal nerve-derived schwannoma in the floor of mouth: a case series

**DOI:** 10.1186/s12903-022-02302-2

**Published:** 2022-06-29

**Authors:** Jiayong Zhong, Zhihang Zhou, Yuhua Hu, Tongchao Zhao, Yu Yao, Laiping Zhong, Dongwang Zhu

**Affiliations:** 1 Department of Oral and Maxillofacial-Head and Neck Oncology, Ninth People’s Hospital, College of Stomatology, Shanghai Jiao Tong University School of Medicine, No. 639 Zhizaoju Road, Shanghai, 200011 China; 2 Shanghai Key Laboratory of Stomatology, National Clinical Research Center of Stomatology, Shanghai Research Institute of Stomatology, Shanghai, China; 3 Department of Oral Pathology, Shanghai Ninth People’s Hospital, Shanghai Jiao Tong University School of Medicine, Shanghai, China; 4 Department of Oral and Maxillofacial Surgery, The First Hospital of Jiaxing Affiliated Hospital of Jiaxing University, Jiaxing, China; 5 Department of Oral and Maxillofacial Surgery, The First Affiliated Hospital of Wannan Medical College, Yijishan Hospital, Wuhu, China

**Keywords:** Schwannoma, Diagnosis, Therapy, Case series

## Abstract

**Background:**

Schwannomas or neurilemmomas are well-encapsulated, benign, solitary, and slow-growing tumors that originate from Schwann cells of the nerve sheath. Extracranial schwannoma is reported to have a relatively high incidence in the tongue while an extremely low incidence in the floor of mouth. In the current study, we presented the first case series of hypoglossal nerve-derived schwannoma in the floor of mouth in Asia.

**Methods:**

A retrospective study of 9 surgical cases of hypoglossal nerve-derived schwannoma in the floor of mouth was performed. The patient and tumor characteristics were evaluated by physical, radiological and pathological examination. Details of operation and complications were also recorded.

**Results:**

Hypoglossal nerve-derived schwannoma in the floor of mouth showed a well-defined boundary with a firm texture, smooth surface and good mobility on palpation. The median maximum diameter of the tumors was 4.3 cm (range 2.8–7.0 cm). The median operative time and bleeding volumes were 89.4 min (range 47–180 min) and 99.2 mL (range 15–200 mL), respectively. All cases received complete surgical excision.

**Conclusion:**

In this study, we presented the diagnosis and management of hypoglossal nerve-derived schwannoma in the floor of mouth for the first time in Asia. The study provided us with a recommendation for consideration of the diagnosis of hypoglossal schwannoma when a patient presents with a mass in the floor of mouth.

## Background

Schwannomas or neurilemmomas are benign and relatively infrequent tumors of the peripheral nerves. It derives from the nerve supporting Schwann cells [1] and grows slowly and painlessly regardless of age or sex [2]. The first description of this type of tumor was made by Verocay in 1910. Although schwannomas are uncommon lesions, approximately 25–40% of extracranial schwannomas occur in the head and neck region [3, 4]. In the oral cavity, the incidence of schwannoma in the tongue is reported to be relatively high; however, it is considered extremely rare in the floor of mouth [5–7]. As schwannomas of the peripheral nerves are relatively infrequent, lesions of the hypoglossal nerve (HyN) are considered to be a rare finding, accounting for only 5% of the non-vestibular schwannomas [8].

In 1998, Drevenlengas et al. firstly described a rare case of hypoglossal schwannoma located in the sublingual space and discussed the differential diagnosis of sublingual mass lesions [9]. In 2009, Fakhry et al. reported a 77-year-old woman with schwannoma of the hypoglossal nerve [10]. However, the principle of therapy and post-operation complications have not been reported in detail [11–13]. Here, we described the diagnosis and management of schwannoma derived from the hypoglossal nerve in the floor of mouth. To the best of our knowledge, it is the first time to report a case series of hypoglossal nerve schwannoma in the floor of mouth in Asia.

## Methods

### Patients and tumors

A retrospective study for the diagnosis and management of hypoglossal nerve-derived schwannoma in the floor of mouth was performed, which included 9 consecutive patients from March 2011 to May 2020. All the Patients enrolled were recruited from Department of Oral and Maxillofacial-Head and Neck Oncology, Ninth People’s Hospital. All the patients underwent surgery for tumor in the floor of mouth. The diagnosis of Schwannoma was confirmed by pathological examination. All the experiments were approved by Ethical Committee of Shanghai 9th Peoples’ hospital. We followed the tenets of the Declaration of Helsinki for research involving human subjects. Information consents were obtained from all the subjects or their legal guardians.

### Diagnosis

#### Physical examination (PE)

The surface of the mass was covered with normal mucosa, and the mass was found with firm texture, smooth surface, well-defined borders and good mobility on palpation.

#### Radiological examination

Complying with routine examination, manganese-enhanced magnetic resonance imaging (MRI) scan was obtained using a 1.5-T imager (Signa, General Electric, Milwaukee, WI). To identify the position and boundary of the tumor, the MRI was performed from clavicle to basis cranii using a T1-weighted spin echo sequence (TR: 500 ms, TE: 25 ms, FOV: 2 cm, depth 1 mm, window width 256 × 192). A T2-weighted spin echo sequence was scanned using the same routine (TR: 3000 ms, TE: 30 ms, FOV: 2 cm, depth 1 mm, window width 256 × 192).

### Pathological examination

The tissue Sections. (4 mm) were stained with H&E staining. Immunohistochemistry of S-100 was detected (primary antibody, dilution 1:200, Abcam Inc.USA; secondary antibody, dilution 1:200, Abcam Inc. USA).

### Treatment and follow-up

The patients underwent a complete surgical resection of the tumor under general anesthesia. A surgical incision was made in the oral mucosa overlying the left Wharton duct, followed by blunt dissection to reveal a membrane-covered lesion. The tumor and the lingual nerve were carefully decorticated; however, there was a branch of the hypoglossal nerve penetrated into the tumor. The tumor and the sublingual gland were clearly demarcated, allowing the duct and the sublingual gland to be preserved. The branch of the hypoglossal nerve was cut, while the trunks of the hypoglossal and lingual nerves were preserved.

After the complete remove of the tumor, a follow-up examination was performed once per 3 months. If any complications or relapse requiring treatment were identified, additional treatment was given until the patient improved or refused to continue treatment.

## Result

### Characteristics of patients and tumors

In this study, 4 males and 5 females were included. The median age was 45.2 years, which ranged from 17 to 63 years. The median maximum diameter of the tumors was 4.3 cm (range 2.8–7.0 cm). There were 8 left HyN Schwannoma and 1 right HyN Schwannoma. Table 1 demonstrated the relative information including definitive histopathological diagnosis, tumor side and tumor volume examined by different method for all the tumors.

**Table 1:** Detail Information of 9 Patients

Case	Age	Gender	Final pathology	Tumor side	Signs and symptoms	Tumor volume evaluated by MR (cm)	Follow-up (month)
1	28	Male	Schwannoma	L	TonD SpeD	3.5 × 2.3 × 2.7	24
2	51	Female	Schwannoma	L	TonD SpeD	4.3 × 3.2 × 2.2	12
3	42	Female	Schwannoma	L	TonD SpeD	7.1 × 4.8 × 3.9	12
4	63	Female	Schwannoma	L	TonD SpeD	2.3 × 3.3 × 4.9	9
5	34	Male	Schwannoma	L	NA	2.3 × 1.3 × 2.3	12
6	17	Male	Schwannoma	L	NA	3.2 × 2.9 × 2.3	12
7	54	Female	Schwannoma	L	SpeD	3.7 × 2.8 × 2.4	6
8	55	Female	Schwannoma	R	TonD SpeD	4.5 × 1.8 × 2.7	12
9	63	Male	Schwannoma	L	NA	2.5 × 2.1 × 1.3	12

TonD. means tongue deviation. SpeD. means speech disturbance

### Surgical process

The surgical details of the 9 cases were shown in Table 2. All patients received surgical treatment underwent general anesthesia via nasotracheal intubation in supine position. All Schwannomas were treated with the intraoral approach. The median operative time was 89.4 min (range 47–180 min). The median bleeding volume was 99.2 mLs (range 15–200 mL). Intermittently suture was performed in closure of wound.

**Table 2:** Detail information of surgery

Case	Approach	OpT (min)	Bleeding volume (mL)	Preservation of sublingual gland	Preservation of branch of the HyN	Preservationof trunk of the HyN	HyN palsy
1	Intra	53	100	P	C	P	NA
2	Intra	105	97	P	C	P	NA
3	Intra	180	100	C	C	C	Ob
4	Intra	69	200	P	C	C	Ob
5	Intra	51	50	C	C	P	NA
6	Intra	93	110	P	C	P	NA
7	Intra	150	171	C	C	C	Ob
8	Intra	47	15	P	C	P	NA
9	Intra	60	50	P	C	C	Ob

Intra. means intraoral cut; OpT. means operation time; Ob. Means observed; P means preseavation; C means cut

### Case presentation

#### Case 1

**Fig. 1:**
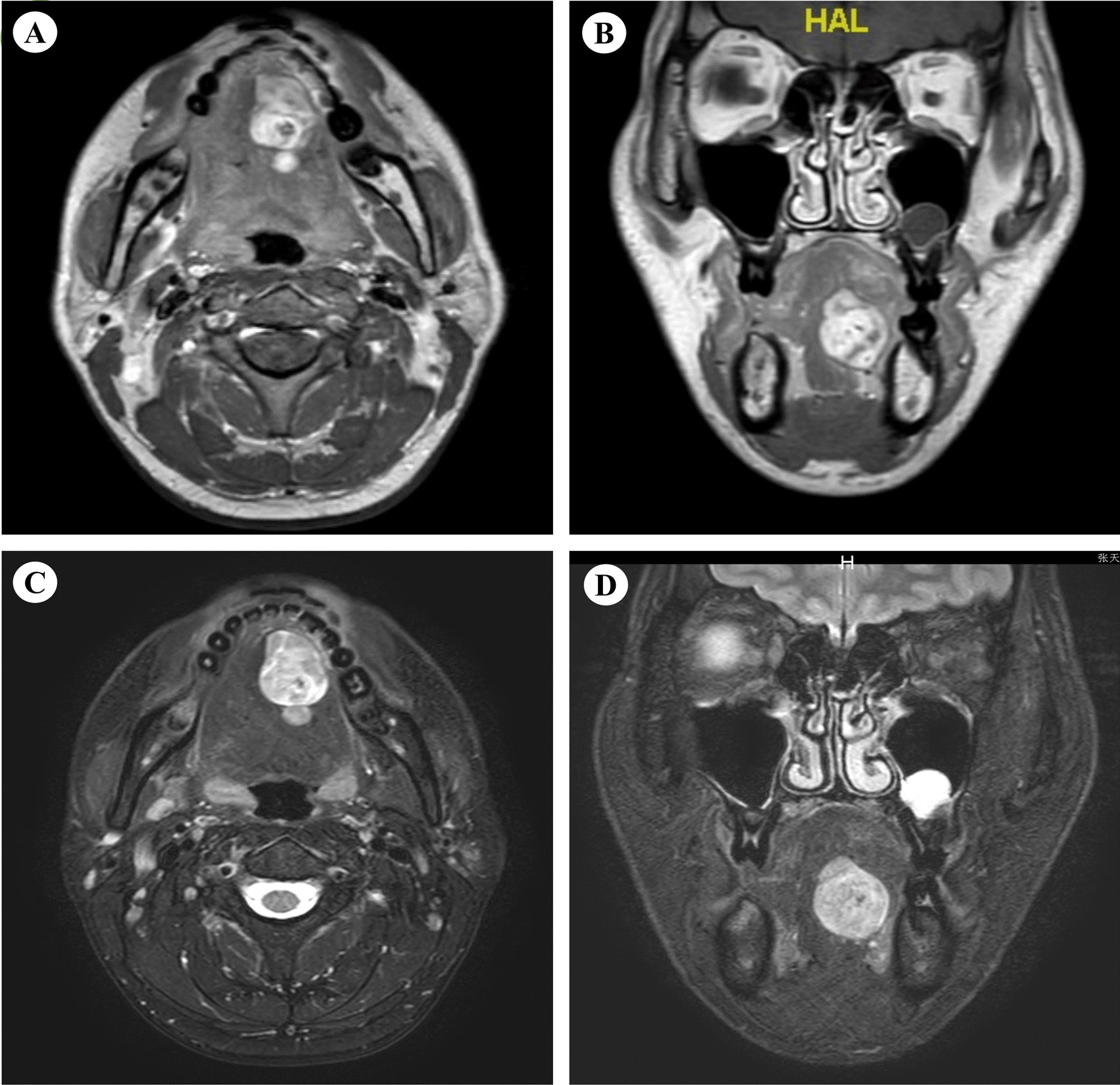
The tumor displayed a heterogeneously equal signal on T1-weighted images (**A**, **B**), and showed a heterogeneously high signal on T2-weighted images (**C**, **D**)

MR images of Case 1 showed an oval mass with well-defined borders occupying the left sublingual space. It was also shown that the extrinsic muscles and the mylohyoid muscle was pushed by oval mass. The tumor displayed a heterogeneously equal signal on T1-weighted images (Fig. 1A, B), and showed a heterogeneously high signal on T2-weighted images (Fig. 1C, D). Depending on MR image, no invasion of the surrounding muscles was observed, which indicated the diagnosis of a benign tumor. According to the findings, the provisional clinical diagnosis was a benign tumor in the left mouth floor. The possibility of a neurogenic tumor was primarily considered.

**Fig. 2:**
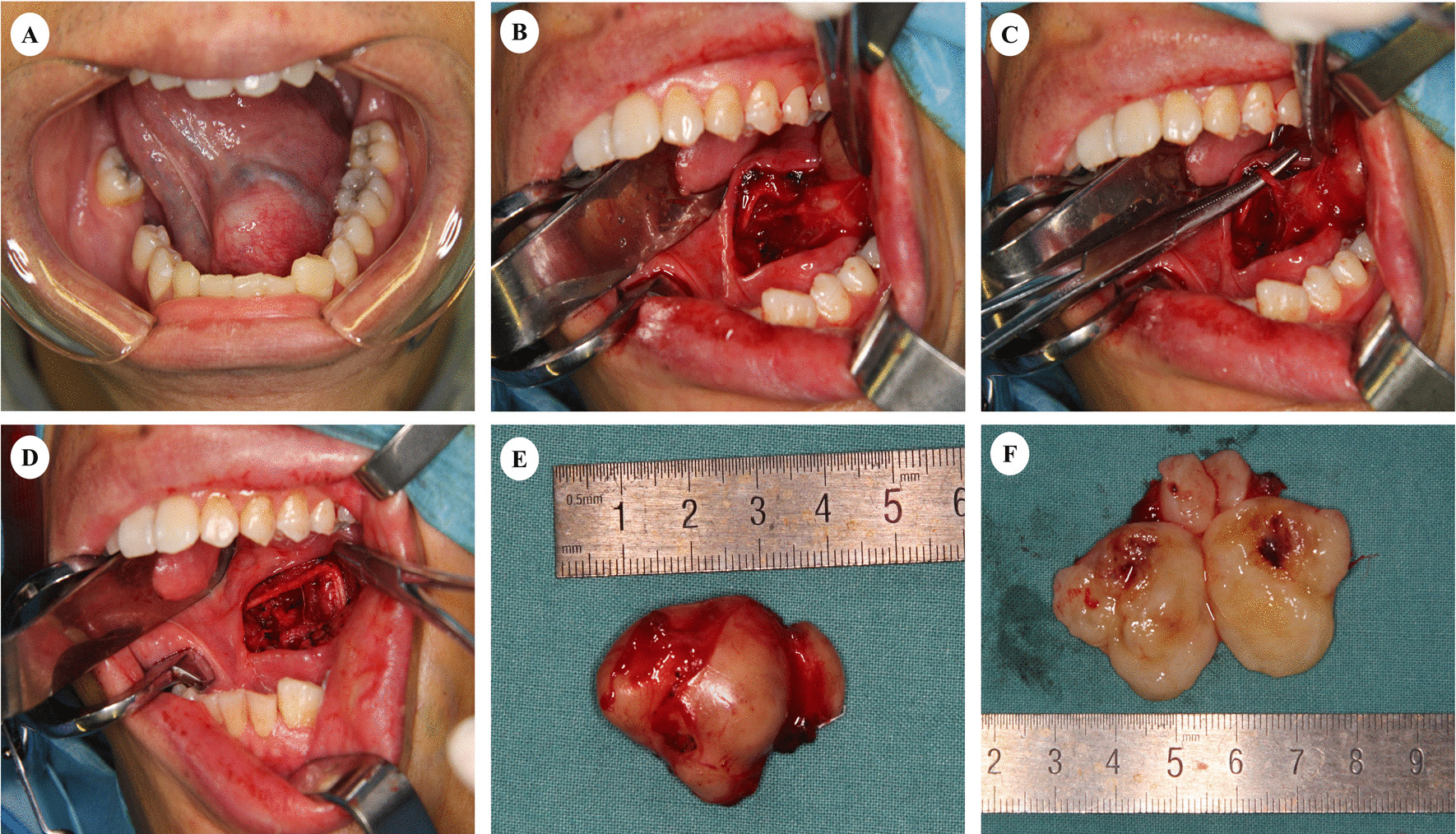
Perioperative clinical photograph. **A**–**D**. The mass was removed through an intraoral approach. **B** The mass was well- encapsulated and removed as a whole. **C**, **D** The tumor and sublingual gland were clearly demarcated, allowing the duct and sublingual gland to be preserved. The branch of the hypoglossal nerve was cut, while the trunks of the hypoglossal and lingual nerves were preserved. **E**, **F**. Postoperative photograph of main mass

According to PE, the surface of the mass was covered with normal mucosa, and the mass was found with firm texture, smooth surface, well-defined borders, and good mobility of the lesion was obtained in the sagittal and coronal direction on palpation. (Fig. 2A).

In Case 1, the branch of the hypoglossal nerve was cut, while the trunks of the hypoglossal and lingual nerves were preserved (Fig. 2B–D). The resected mass was 4.2 × 2.5 × 3.5 cm in size, yellow in color, encapsulated, oval shape, smooth and firm in consistency (Fig. 2E, F). There was no numb in the left side of the tongue and no tongue deviation after operation. During the follow-up period, no recurrence of the tumor has been observed.

**Fig. 3:**
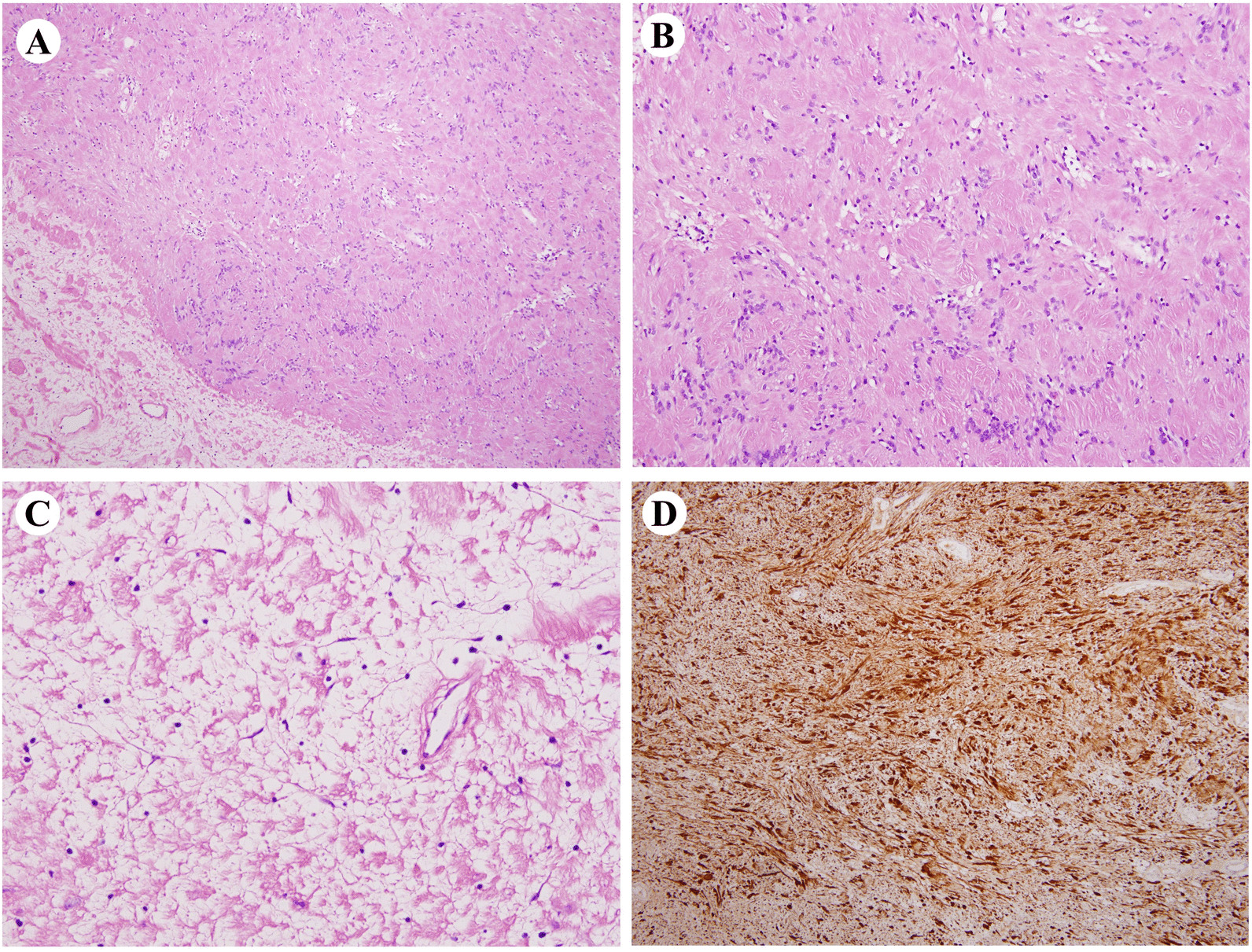
**A** Pathological examination of the mass. **B** Antoni A pattern with well-organized, high cellularity, right (H&E staining, × 40). **C** Antoni B pattern with less cellularity, left. (H&E staining, × 40). **D** Immunohistochemistry shows reactivity to S-100 protein (×40)

Pathological examination revealed a well encapsulated tumor exhibiting areas of organized spindle-shaped cells in a palisading arrangement around acellular, and eosinophilic areas forming Verocay bodies giving Antoni type ‘A’ pattern. Other areas with Antoni type ‘B’ pattern exhibited less cellularity with less organized cells, which were plump, spindle-shaped and were generally seen adjacent to dense vascular areas (Fig. 3A–C). Immunohistochemical investigation of the tumor cells showed diffuse, strongly positive staining of S-100 protein (Fig. 3D). These findings were compatible with the diagnosis of Schwannoma.

## Discussion

Former studies[14–16] independently reported the mean patient age of 42, 32.6 and 45 years respectively. The epidemiologic data onto the patients included in this study match previously description of gender and age distributions. Most of the literature reported the most common site of extracranial schwannomas in the head and neck is the parapharyngeal region [17], with floor of mouth localization, especially derived from HyN, being relatively rare.

Clinically, schwannomas are often misdiagnosed as other common benign lesions such as pleomorphic adenomas, fibromas, or mucous retention cysts on account of their slow growth. Patients usually present with an insidious-onset gradually progressive swelling which may or may not be accompanied by paresthesia [17]. The most common presenting symptoms of Hyn-derived schwannoma included tongue deviation [18], headaches [19], vertigo and nausea [11]. Hypoglossal nerve palsy was the most frequent presenting sign, occurring in 80% of the cases [20]. In our study, we limited the location to the mouth floor. Accordingly, the symptom of speech disturbance and tongue were noted in 66.6% and 55.5% respectively, while other symptoms were not recorded.

MRI was by far the most frequently utilized imaging modality used for the auxiliary diagnosis of schwannoma. Previous studies indicated that hypoglossal schwannomas appear T1 hypointense and T2 hyperintense, with heterogeneous enhancement in contrast-enhanced studies [21]. In case.1 of our study, the tumor displayed a heterogeneously equal signal on T1-weighted images, and showed a heterogeneously high signal on T2-weighted images. The hyperintensity of hypoglossal schwannomas on T1 images can be variable depending on the predominance of Antoni B fibers [22].

Schwannomas are highly radio-resistant; therefore, radiotherapy is not indicated for their management [13]. The surgical excision is recommended as the primary therapeutic strategy for hypoglossal schwannomas. Considering the high morbidity and mortality rates before 1970, the standard treatment for neurilemmoma is complete surgical excision [23–25]. The malignant transformation of schwannomas is extremely rare [26]. Outcomes after surgical excision of hypoglossal schwannomas tend to be favorable. Only two cases [27, 28] prior to 1970 ended in death were reported. In our research, no recurrence of the tumor has been observed after follow-up.

When it comes to hypoglossal schwannomas in mouth floor, intraoral approach was performed in formal studies [9, 10]. The intraoral approach remains the first choice to resect such tumor, and once removed completely, schwannomas do not recur. Identifying the nerve of origin may be difficult, as it is difficult to differentiate between tumors of the lingual, hypoglossal and glossopharyngeal nerves [29]. Some studies recommended that intraoperative neuromonitoring, particularly of the 10th and 12th cranial nerves, can be considered during surgery [30]. In our research, the mass was infiltrated by a branch of the HyN, which had to be sacrificed for complete excision of the tumor, no HyN palsy was observed immediately after surgery.

Depending on the pathological results, schwannomas is characterized as an benign tumor with encapsulated or cystic structures. Schwannoma has consisted of spindle-shaped cells, which was arranged in two different types: Antoni A and Antoni B patterns; where Antoni A type refers to a densely packed pattern of cellular arrangement, while Antoni B represents a more loosely arranged pattern. The axons of the underlying nerve are usually stretched over the tumor capsule. Moreover, immunohistochemistry of S-100 protein was selected to identify schwannoma[12]. The tumor presented in the current report showed characteristics typical of schwannomas.

## Conclusion

In this research, we reported a Case series of hypoglossal nerve-derived neurilemmoma in the floor of mouth for the first time in English language literatures. The research provided us a recommendation for consideration of the diagnosis of hypoglossal neurilemmoma when a patient present with a mass in the mouth floor.

## Data Availability

The datasets usded and analysed during the current study are available from the corresponding author on resonable request.

## Abbreviations

PE: Physical examination
HyN: Hypoglossal nerve
MRI: Magnetic resonance imaging
H&E: Hematoxylin and eosin
